# UK physician associate primary care placements: staff and student experiences and perceptions

**DOI:** 10.5116/ijme.5bcf.7914

**Published:** 2018-10-31

**Authors:** Rhiannon Hoggins, Wesley Scott-Smith, Michael Okorie

**Affiliations:** 1 Brighton and Sussex Medical School, Brighton, UK; 2 Department of Medical Education, Brighton and Sussex Medical School, Brighton, UK

**Keywords:** Physician associates, students, education, primary health care, general practice

## Abstract

**Objectives:**

To provide an insight into the experiences and perceptions of physician associate students and primary care staff involved in primary care educational placements in the United Kingdom.

**Methods:**

A qualitative study was conducted. Data were collected from focus groups and semi-structured interviews with eight first year physician associate students and six primary care staff in two general practice surgeries in East Sussex, United Kingdom. Recruitment was via purposeful sampling. Thematic Analysis was used to identify themes.

**Results:**

Three themes were identified: perceptions of the physician associate role, interprofessional working, and the physician associate course structure and placements. Staff demonstrated a lack of familiarity with the physician associate programme and there was a risk of unrealistic expectations. Overall, staff and students were positive about their experiences. However, students expressed anxiety over a large amount of learning in a short timeframe, the perceptions of others, and the reluctance of staff to train them in phlebotomy skills. In addition, students were unsure about their career aspirations for the future.

**Conclusions:**

Participants were positive about their experiences however students expressed a number of anxieties, with a scope to improve interprofessional education. Practice staff demonstrated an overall lack of knowledge of the curriculum and physician associates in general leading to a risk of unrealistic expectations. Further studies on these themes with a larger sample size across relevant training institutions in the United Kingdom is required to explore this further.

## Introduction

Primary care is facing increasing demand and an increased workforce burden.^1^^,^^2^ Despite this, the number of junior doctors choosing to become general practitioners (GPs) is not commensurate.^1^^,^^2^ These challenges have identified the need for more appropriate working practices and underpinned the introduction of physician associates (PAs) in the UK in 2003. This role is closely related to the role of physician assistants in the United States of America, which was introduced in the 1960s. As of June 2017, there were 450 PAs and 1200 PA students living and working in the UK.^3^

In 2016 Brighton and Sussex Medical School (BSMS) admitted its first cohort of PA students. This two-year postgraduate course incorporates 1600 hours of clinical placement which is divided amongst a variety of medical specialities, and during the first academic year, the course entails a longitudinal placement solely in primary care over 9 months. Furthermore, it is anticipated that a large proportion of the PA students will be employed in primary care after qualification since evidence suggests that the three largest areas of PA employment currently are all generalist, encompassing general practice, acute medicine, and emergency medicine.^4^^,^^5^

In 2012 the UK Department of Health published the Competence and Curriculum Framework (CCF) for physician associates which defines the curriculum and competencies which all PA courses must adhere to in the UK.^6^ This document has considerable overlap with ‘Promoting Excellence’ published by the General Medical Council (GMC).^7^ The CCF works on a symptom-based rather than disease-based format. Portfolio requirements for final year students are very similar between PAs and undergraduate medical students; using similar clinical reasoning methods. Furthermore, the training of PAs is based on the same biomedical model of health as doctors. This model focusses on the physical or biological aspects of disease and illness; associated with the diagnosis, cure, and treatment of disease.^8^

A review of the literature was performed using search terms 'physician associate', 'primary care' and 'teaching programme’; using Boolean operators ('AND', 'OR' and 'NOT') to combine these search terms, as well as synonyms and subject headings for each key part of the research question. An asterisk was also used to truncate certain words. This review indicated that there is a complete dearth of evidence published in PA primary care education. There are a number of studies published in the UK which focus on PAs working in primary care. Williams and Ritsema surveyed 61 doctors representing 14 specialities or medical settings in the UK.^9^ They explored the perceived benefits and challenges of the role of PAs from both the doctors’ and patients’ perspective. Their findings suggest that British doctors were generally satisfied with the PA role and patient feedback was mainly positive. However, this was a limited sample of doctors, and none of these studies focus on the PA students' education.

Only one paper was found internationally which focussed on PA students' teaching during placement in primary care, and Kayingo and colleagues conducted this in the USA.^10^ The study aimed to explore the PA students' roles and satisfaction with their primary care rotations. Students worked with a variety of healthcare professionals (HCPs), with their favourite parts of the placement being their clinical responsibilities and interactions with other clinicians. Their least favourite parts were the quality of the formal teaching sessions. However, this study used a survey to collect primarily quantitative data. Other published research studies have focussed on specific topic areas of the PA's teaching; for example, neurology and oncology, but these do not focus on primary care.^11^^,^^12^

The PA role is a relatively new role in the UK, with an increasing number of PA educational programmes now being delivered. Since the literature review has shown that there have been no qualitative studies conducted internationally surrounding the PA student education programmes, this study aimed to explore the PA students' experiences and staff perceptions of the primary care placement for PA students in the UK. This is in an attempt to optimise teaching and learning of PA students in primary care. By using a qualitative approach, this study will provide a unique insight into PA students' primary care education.

## Method

### Study design

An exploratory qualitative study was conducted using thematic analysis. Qualitative methodology was used since this study focuses on the experiences and perceptions of both the PA students and the primary care staff; highlighting how this is affected by the social interactions between the PA students and the staff and therefore how their behaviour might be influenced.

### Sampling

Purposeful sampling of primary care staff from the participating GP practices was performed in order to include a variety of HCPs and administrative staff.  Focus groups and interviews were used to allow for the collection of rich, detailed information from the participants. The PA students and primary care staff were recruited separately via email. The inclusion criteria for this study were either first-year PA students studying at the medical school or staff involved with the PA primary care placement programme who are working at the primary care sites involved with the school.

### Recruitment of participants and setting

Eight first-year PA students (five females and three males) studying at BSMS and six primary care staff (five females and one male) were recruited. The six primary care staff were based across two GP surgeries in East Sussex, UK. There were two GPs, one foundation year two doctor, one practice manager, one deputy reception manager, and one healthcare assistant recruited.

### Data collection

The email addresses of the PA students and the lead GP mentor at each primary care site in East Sussex were provided by the course lead and administrator. The students and staff were emailed directly using the BSMS email system inviting them to participate in the study, with the participant information leaflet, consent form and contact details for further information attached. We also spoke to the students at their lectures to invite them to participate. The lead GP mentor was also asked to further distribute the email to other members of staff at that practice. Data collection was via two methods which included two focus groups comprising four PA students each and face to face semi-structured interviews with six primary care staff.

In the case of the PA students, data were gathered by focus groups which were audio-recorded, and these were conducted at BSMS. Two focus groups were conducted with four participants randomly allocated to each group. Staff data were gathered from face to face semi-structured interviews which were audio-recorded and these were conducted at the primary care sites in East Sussex.

Data were collected on the participants' perceptions and experiences of the primary care placement programme and the PA educational curriculum. There was also focus on the role of the PA in primary care (including how this fits into the UK National Health Service (NHS) workforce) and the state of primary care currently in the UK, as well as PA regulation and career aspirations for the future.

### Qualitative data analysis

Thematic analysis, independently triangulated (audio-recordings were transcribed verbatim) by the research team was used to analyse the data from the interviews and focus groups and validated through theoretical saturation across all participants. The verbal data were transcribed into written form, and then we proceeded to familiarise ourselves with the data. While doing this, notes were made in the margins of the transcripts; noting any initial ideas and therefore generating a possible list of themes. This was done separately for both the student transcripts and the staff transcripts. Once this process was complete it was important to consider which themes were theoretically saturated across the participants; whilst doing this it was helpful to produce spider diagrams using post it notes. Concepts were first labelled as the "first iteration" of the data which demonstrate relatively concrete terms. In comparison, those in the "third iteration" demonstrate the transition towards a perspective which explains the 'whole process', influenced by other ideas. Whilst transitioning from the first to the second iteration it was essential to evaluate which themes were theoretically saturated.

The themes that were labelled according to the degree of theoretical saturation (across both groups and individuals) were as follows: perceptions of the PA role; interprofessional working; the PA course structure and placements. Each of these will be discussed below, using illustrative saturated quotes to represent the themes of the data.

During this study it was possible that participants may experience emotional distress. The appropriate support was available via BSMS should this have happened. We were aware of this possibility and were prepared to signpost them to the appropriate support. This study was approved by the Brighton and Sussex Medical School Research Governance and Ethics Committee (RGEC).

## Results

Using thematic analysis, three themes were identified:  perceptions of the PA role, interprofessional working, and course structure and placements. These themes are explored in detail as follows:

### Perceptions of the PA role

Students and the staff discussed their perceptions of the primary care placement programme and the role that PAs can have in primary care. Across all of the participants, there were discrepancies surrounding the perception of the role of PAs working in primary care in the future. Both the staff and the students felt that the PAs would have a useful and varied role whilst working in primary care, including minor illnesses, clinics, and home visits. They felt that this would evolve over time as the GP practices begin to develop trust in the PAs, and would depend on the PAs’ experiences and interests. Staff felt that all of this should be in line with the PAs' competencies and level as outlined by the CCF.

"...I think you know you can just build it up through trust… so you might start off with minor ailments or long-term illnesses but then you can see more complicated things" (No 5, Female, PA student)

"...we can try and reduce the pressure and then doctors can actually focus on things" (No 7, Female, PA student)

 “...once you find out what level the PAs can work at; what specialities they can see...then we can tailor a clinic towards them" (No 1, Female, Practice manager)

Both the students and the staff reported feelings of uncertainty around what to expect of the PA students. Some students felt that the staff did not understand the PA role or had a lack of knowledge of the PA curriculum (including the PA matrix and the medical model) and as a result had unrealistic expectations. Furthermore, some of the staff agreed with this; expressing that they felt that they did not know what to expect of the PAs initially due to their own lack of knowledge.

"I think at the beginning I found it more difficult. I think the role wasn't quite understood and expectations weren't quite understood of what we were meant to do" (No 5, Female, PA student)

"...I don't think it had been properly outlined to the to the practice what the course expected from them" (No 8, Male, PA student)

"We weren't really educated into what was expected when they first came...I think if we had had a bit more knowledge about their level of competence" (No 1, Female, Practice manager)

The CCF incorporates the PA Matrix which is a model specifying the core clinical conditions for the PA by category of level of competence. University educational programmes base their curriculum on this matrix. In this matrix, conditions are ranked as "1A, 1B, 2A or 2B" which represents the level of knowledge required about each condition.

In order to overcome these difficulties, the students felt that the PA role and level of the students' clinical experience needed more explaining to the staff at the start of the placement. They suggested that this included more meetings between the BSMS faculty and the GP supervisors, as well as more meetings between the GP supervisors and the staff working at each individual practice.

"...I had to educate him really in what I can do, what I do know...his expectations I think had to come down a bit" (No 1, Female, PA student)

"Yeah it's just about educating people about the role" (No 7, Female, PA student)

"...yeah I think any education for the practices in what we can do certainly is an essential part of the organisation..." (No 8, Male, PA student)

Many of the students exhibited feelings of anxiety related to a number of aspects of their training and their role as a PA in the future. This was because it was a new role integrating into the NHS. However, students believed that there were potential solutions to this; the central point being communication.

"Definitely. It's a new role it's going to happen. I think it's just as I said, communication…I think if the roles properly explained to them..." (No 5, Female, PA student)

"...in the practices that we're in at the moment, if I was to judge based on that I would say it's excellent...we can already see that there's a lot of different opinions from different practices, so I know that I will probably come into contact with some issues" (No 7, Female, PA student)

Students were also anxious about their current lack of knowledge and the need to progress quickly as a student PA. The UK PA university course is based on the five-year undergraduate medical school curriculum; however, it is very condensed, with the students training to be a PA for two years following their undergraduate degree.

"There has to be a point where you think "well I actually have to do this job"…when I come out of this after being a student they're going to expect me to do it. We've only got four, five months of teaching left…it's not like I'm going to learn everything next year...I need to learn now" (No 2, Male, PA student)

"...it's a bit nerve-racking...I definitely don't feel ready at the moment" (No 3, Female, PA student)

Many of the students were anxious about the media's perception of PAs in the future and were concerned that this would reflect poorly on them. To date, there have been a number of newspaper articles published focussing on PAs working in the UK with some not always reflecting positively on this profession.^13^

"All it would take is for a PA to go out there and something bad to happen and it would be all over the press…" (No 2, Male, PA student)

"They call us the junior doctors on the cheap..." (No 5, Female, PA student)

"...the only thing I fear is a sort of a negative media impression which may come out of the blue all of a sudden... and I wholeheartedly expect it at some point" (No 8, Male, PA student)

Students and staff reported positive experiences and perceptions in relation to the PA primary care placement programme. The staff felt that it was good for the PA students to come into the practice as part of their learning and that they could help the practices; overall they were extremely positive about the PAs.

"…we wanted to support the physician associates. We really feel that there will be a place for them in primary care" (No 1, Female, Practice manager)

"it's great that that such a big chunk of their training is in primary care...it's been very positive...I've enjoyed having them" (No 2, Female, GP)

"...he's extra hands now...so that was really useful. If you get that student coming to us qualified putting him into a role I think, excellent!" (No 6, Female, Healthcare assistant)

Similarly, on the whole, the students had positive experiences of their primary care placement. During the interviews they reported enjoying the placement, learning a lot and a smooth transition to their placement in primary care.

"I've got a fantastic practice...I've been very fortunate to be where I am" (No 2, Male, PA student)

"I'm learning a lot. I really enjoy going to my GP practice" (No 3, Female, PA student)

"...my personal experience so far has been very good...I've been quite lucky with my practice because everything's gone very smoothly" (No 7, Female, PA student)

Due to the current pressures on general practice, the UK government are keen to recruit PAs into primary care surgeries that are local to where the students have trained. However, the students demonstrated differing career aspirations in relation to working as a PA in the future; whilst reporting that they had enjoyed working in primary care, they expressed that they were yet to experience secondary/tertiary care and therefore they were unsure where they would like to work in the future.

“... I quite like primary care but I haven't worked in hospitals so I don't really have a feel for it" (No 4, Male, PA student)

"...I would like to go down the surgical route to begin with and then eventually end up in primary care" (No 5, Female, PA student)

"...so far I can see myself in primary care but I haven't experienced secondary care yet so can't really visualise where I'll be" (No 6, Female, PA student)

### Interprofessional working

Students and staff reported that the students interacted with a wide variety of HCPs and staff. This included GPs, junior doctors, healthcare assistants (HCAs), nurses, paramedic practitioners, administrative and reception staff. This was in order to experience a range of clinical practices, methods of working and opinions. This allowed them to observe the day-to-day running of a GP surgery and the multidisciplinary team approach which takes place.

"...I've been with the nurses and the GPs. With a mixture of, not always just one nurse, or the same nurse, or the same GP. Which is nice to see other peoples' takes on how they do things differently" (No 1, Female, PA student)

"GPs, nurses of various grading, healthcare assistants...spent a little bit of time with a paramedic practitioner but that's pretty much it" (No 3, Female, PA student)

"...pretty much all of them... they've spent some time sitting in with the healthcare assistants... nurses... admin... reception" (No 3, Male, Practice manager)

Students and the staff reported minimal interaction between the PA students and other healthcare professional students as part of the primary care placement. Occasionally students would meet over coffee break; however, this was the extent of the interprofessional interaction between these students. Interprofessional education (IPE) is an essential aspect of any healthcare professional students' training in order to raise awareness of other team members' roles and especially in this case, the role of new team members.^14^^,^^15^ There was often little IPE between the PA students and other HCP students because the students did not tend to visit the GP practices on the same days or did not have other students attending the practices at all. There is scope to improve this in the PA programme in the future.

"No, we don't see them...I know that they have them but I think on different days" (No 3, Female, PA student)

"...I'd say no. Cos we've interacted with them as in talked to them but only over coffee break...nothing more than that" (No 5, Female, PA student)

"...they haven't sat in with them, but there's been discussion over coffee" (No 2, Female, GP)

### Course structure and placements

The organisation of the primary care placement programme was a key theme raised by both staff and students. This focussed on a number of aspects of the placement. Since the PA educational programme is a postgraduate course, there is an increased emphasis on independent learning. Students discussed how during their spare time they would study further than what they had been taught during their formal teaching; using the PA Matrix as a source of revision. However, many of the students felt that they had too much time off for independent study and would like more formal teaching, such as lectures.

Staff and the students reported that the PA students engaged in a wide range of clinical activities, and these included: taking histories and practicing examinations, minor practical procedures, observing clinics, as well as more invasive procedures such as phlebotomy. This is in line with the BSMS curriculum as detailed in the student workbook, as well as the CCF and PA Matrix.

"...seeing patients and examining them” (No 2, Female, GP)

"...minor practical procedures like throat swabs, requesting urine...or sometimes if there's an interesting examination sign like listening to chests" (No 2, Female, Foundation year two doctor)

"... past two or three months we've just been taking histories over and over and over" (No 5, Female, PA student)

A proportion of the participants reported difficulties surrounding the students engaging in phlebotomy in clinical practice. The nurses and HCAs were concerned about having adequate protection from their employers should there be any issues that might arise as a result of training the PA students in phlebotomy. The students who were completing their clinical placement at this practice perceived their lack of phlebotomy training in practice to be a negative; concerned that this may become a deficiency in their skill set.

"…we right at the beginning said "no we're not teaching you to take blood” …they had some problems with the nurses feeling competent to sign them off. I've spent some time and our manager has spent some time talking to the nursing staff to try and reassure them that if I am happy to sort of protect them" (No 2, Female, GP)

"...I do feel like it's a shame that we can't do clinics because I feel like my skills are becoming a bit deskilled in it" (No 5, Female, PA student)

"...there was this sort of reluctance; basically the HCAs didn't feel comfortable in saying we could sign you off" (No 8, Male, PA student)

## Discussion

Overall the students and staff were positive about their experiences of the PA student primary care placement programme. Both groups of participants perceived that PAs will have a significant role in UK healthcare delivery in the future. However, the students did express a level of anxiety over the requirement of a large amount of learning in a short period of time and the perceptions of PAs by other HCPs and the media.^13^ It is important that the students' concerns surrounding the perceptions of other HCPs and the media is addressed by faculty in the future by reassuring the students, as well as the faculty interacting effectively with primary care staff in order to promote the role of the PA.

Many of the staff demonstrated an overall lack of knowledge of both the PA student curriculum and PAs in general, and there is a risk of unrealistic expectations. In the future, these observations should be addressed at the start of each PA student cohort to ensure that the lead GP mentor and other primary care staff receive enlightenment in these aspects; including information on litigation. Students engaged in a wide range of clinical activities, however, there was some reluctance to train students in phlebotomy due to a lack of clarity around the regulation of PAs.

Students interacted with a variety of primary care staff, but there is some scope to improve IPE with students of other HCPs. Increased IPE with other students may be useful to promote the role of the PA and to improve multi-professional working with other healthcare professionals; this may help to relieve some of the PA students' anxieties.^14^^,^^15^

### Comparison with existing literature

This is the first study in the UK to highlight the student experience and staff perceptions of primary care educational placements for PA students. These findings can be compared to research conducted by Williams and Ritsema who surveyed 61 doctors in the UK who had worked with a PA and demonstrated similar results to this study surrounding the positive perceptions of the PAs.^9^  Furthermore, in a similar way to this study they reported that doctors were concerned that the unregulated status of PAs could put them and the PAs in a legally compromising situation. However, it is important to note that participants surveyed by Williams’ and Ritsema were all doctors, whereas in this study we have interviewed a variety of staff members (such as a practice manager, a receptionist, an HCA, and doctors). Therefore, the results can not necessarily be directly compared.  Furthermore, the researchers in the named study did not focus on PA students or their education. The findings of this research project can also be linked to the study in the USA, published by Kayingo et al. who evaluated the training of PA students engaging in a primary care teaching programme.^10^ This quantitative study covered teaching methods, clinical activities, interprofessional learning, and career aspirations. Similar to our study, Kayingo et al. found that students interacted with a variety of HCPs. Furthermore, our study results suggest that PA students engaged in a wide range of clinical activities, and interacted with a variety of different HCPs as was the case in the Kayingo et al study. However, it is important to note that Kayingo et al. explored the PA students' roles and satisfaction using a quantitative research approach and so the results may not be directly comparable with that of this study. 

### Limitations of the study

Limitations of the study include the small sample size: only eight PA students and six members of primary care staff volunteered. In addition, the results of this study represent the views of individuals involved in a primary care placement within one UK medical school only, and therefore is a single-centred study. As such, the results are unlikely to be fully representative of the target population and therefore may not be generalisable. Furthermore, some of the participants may have been less likely to voice their opinions in a focus group compared to others. This could be overcome in future studies by performing face to face interview with the students.

The potential for bias is acknowledged. However, in order to minimise the risk of bias triangulation of data with other members of the research team was performed.

## Conclusions

This research project has elicited the experiences and perceptions of both the PA students and the staff, and has allowed for comparisons to be made between the two groups. Furthermore, it has enabled reflection to occur on the implications of these findings on future PA teaching programmes and clinical practice in the UK. This study has shown that participants were positive about their experiences whilst expressing a number of anxieties. These anxieties need to be addressed in the future to ensure that students can learn in a positive and welcoming environment. Staff demonstrated an overall lack of knowledge of the curriculum and PAs in general, leading to a risk of unrealistic expectations. There is scope to improve interprofessional education with other groups of students, and this may help to address the students' anxieties about the perceptions of their role by others and the unrealistic expectations demonstrated by other HCPs. By tackling the primary care mentors' unrealistic expectations of the students, the PA students' overall medical education experience could be augmented, with increased alignment between the university curriculum and their clinical exposure.

Further research using a larger sample size across other medical schools in the UK is required to confirm similar experiences in areas that have employed physician associates for longer periods, and therefore might have developed a different perspective upon the role. This could be performed using a questionnaire based on the themes developed during this study and could be distributed across UK programmes. Interviewing teaching faculty in medical schools would be useful in order to compare and contrast their experiences, as well as extending the survey to graduate PAs and patients.

### Conflict of Interests

The authors declare that they have no conflict of interest.

### Acknowledgements

The authors would like to thank the students and primary care staff who participated in this study.
